# Enabling Superhydrophobicity-Guided Superwicking in Metal Alloys via a Nanosecond Laser-Based Surface Treatment Method

**DOI:** 10.1021/acsami.1c09144

**Published:** 2021-08-20

**Authors:** Avik Samanta, Wuji Huang, A. S. M. Sazzad Parveg, Parth Kotak, Raymond C. Y. Auyeung, Nicholas A. Charipar, Scott K. Shaw, Albert Ratner, Caterina Lamuta, Hongtao Ding

**Affiliations:** † Department of Mechanical Engineering, University of Iowa, Iowa City, Iowa 52242, United States; ‡ U.S. Naval Research Laboratory, 4555 Overlook Ave., SW, Washington, D.C. 20375, United States; § Department of Chemistry, University of Iowa, Iowa City, Iowa 52242, United States

**Keywords:** superwicking, surface functionalization, superhydrophobicity-guided, laser surface texturing, chemical modification, capillary

## Abstract

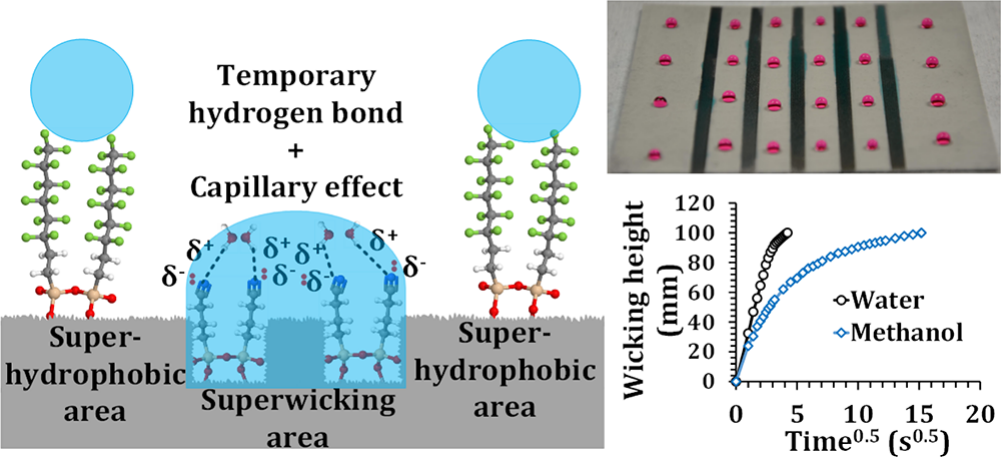

Enabling capillary wicking on bulk metal alloys is challenging due to processing complexity at different size scales. This work presents a laser-chemical surface treatment to fabricate superwicking patterns guided by a superhydrophobic region over a large-area metal alloy surface. The laser-chemical surface treatment generates surface micro/nanostructures and desirable surface chemistry simultaneously. The superhydrophobic surface was first fabricated over the whole surface by laser treatment under water confinement and fluorosilane treatment; subsequently, superwicking stripes were processed by a second laser treatment in air and cyanosilane treatment. The resultant surface shows superwicking regions surrounded by superhydrophobic regions. During the process, superwicking regions possess dual-scale structures and polar nitrile surface chemistry. In contrast, random nanoscale structures and fluorocarbon chemistry are generated on the superhydrophobic region of the aluminum alloy 6061 substrates. The resultant superwicking region demonstrates self-propelling anti-gravity liquid transport for methanol and water. The combination of the capillary effect of the dual-scale surface microgrooves and the water affinitive nitrile group contributes toward the self-propelling movement of water and methanol at the superwicking region. The initial phase of wicking followed Washburn dynamics, whereas it entered a non-linear regime in the later phase. The wicking height and rate are regulated by microgroove geometry and spacing.

## Highlights

- Laser-chemical surface treatment creates a patterned superwicking-superhydrophobic surface.
- Superhydrophilic chemistry inside the superwicking region enhances wickability.
- Superhydrophobic surroundings restrict the sideways liquid flow and guide the flow vertically.
- The narrower gap between microgrooves and high aspect ratio microgrooves provides enhanced wickability.

## 1 Introduction

Superwicking is generally assigned toward a solid surface with a high affinity toward a liquid so that the liquid immediately disperses on the surface upon contact. In recent years, wicking transport of liquids in porous media has received significant interest in both fundamental understanding and application front. There is significant complexity involved in transport mechanisms that support different length scales as capillary-driven liquid transport favors a small length scale, whereas viscosity restricts liquid movement in a small length scale.^1−4^ Hierarchical porous morphologies containing multiple length scales are beneficial for mitigating the restrictions imposed by viscosity and concurrently optimize each transport phenomenon.^5,6^ The wicking behavior in porous structures performs a significant role in several applications, including moisture harvesting,^7−9^ thermal management,^4,10^ microfluidics,^11^ biomedicine and dental treatment,^12^ fuel cells,^13^ biomedical devices,^14,15^ and heat pipes.^16−18^

The final wettability and wicking behavior of a surface depend on the combination of solid and liquid properties.^19−21^ Therefore, wicking behavior is expected to be influenced by three major factors: (i) solid surface chemistry, (ii) solid surface morphology, and (iii) surface tension and viscosity of liquid. During the wicking process, liquid permeates into the surface structures, and liquid penetration follows a diffusion relation where the penetration distance is proportional to the square root of time, as predicted by Washburn.^1^ The wicking behavior on silicon micropillars showed that the liquid penetrates the surface structures, and the penetration depth is decided by the balance between the viscous force of the liquid and capillary force around the micropillars.^22^ Photosensitive oxide semiconductor ZnO nanowires^23,24^ were also used for wicking research, where UV illumination enhances the wicking activity by influencing the surface chemistry. UV illumination generates surface oxygen vacancies in ZnO, resulting in adsorption of the water molecules, leading to excellent superwicking behavior.

Over the years, different engineering methods were investigated to create geometrical features for wicking dynamics. Those wicking surface topographies can be categorized into two major groups: (i) single-length scale with homogeneous porous media^23−27^ and (ii) dual-scale roughness.^5,6,28,29^ Single-length scale surface topographies include vertical arrays of synthesized nanotubes^26^/nanowires,^23,24,27^ nanofiber coating,^25^ and patterned nano/microstructured pillars by deep reactive ion etching.^30^ Water spreading and wicking activities were investigated on a forest of carbon nanotubes on the zircaloy surface in terms of the amount of absorbed water and wicking speed.^26^ For wicking enhancement, the solid surface was covered with silicon nanowires.^27^ In these cases, wicking dynamics was improved by increasing the capillary pressure due to the high surface roughness of nanowires. Excellent moisture wicking was achieved by two layers of nanofibers, including hydrophilic polyacrylonitrile (top layer) and hydrophobic polystyrene (bottom layer), coated by electrospinning.^25^ However, a more significant viscous force is generated with smaller surface features and narrower pores, which hinders the flow for liquids with high viscosity. Consequently, highly roughened surface nanostructures do not automatically result in improved wicking activity due to viscous resistance. Dual-scale hierarchal structures provide better provision for wicking activity for high viscous liquids. Wang et al. fabricated 3D dual-scale ZnO nanopillars covered with ZnO nanowires using interference lithography and hydrothermal synthesis and improved the wicking properties by up to a factor of 3.^28^ Charlton et al. fabricated hierarchical surface structures with silicon nanopillars using reactive ion etching along with the second level of silicon oxide roughness using room-temperature plasma-enhanced chemical vapor deposition.^6^ Lee et al. fabricated vertical CuO nanocactuses comprising submicron nanowires covered with sharp nanoscale oxide features. The hydrophilic nature of CuO nanocactuses resulted in enhanced capillary wicking.^5^

Extreme wettability patterns represent surfaces encompassing contrasting wetting regions consecutively in a designed orderliness,^31^ and their properties enable the control of fluids in specific wettability segments. Most of the existing fabrication processes require multistep processing using masks for selective treatment of extreme wetting areas.^32−35^ Electrochemical etching was used to selectively create the surface structure in the specified area followed by selective surface chemistry modification.^33−35^ External UV illumination-based techniques were developed to fabricate patterned extreme wetting surfaces in recent years^36−38^ based on photocatalytic decomposition under UV illumination.^39^ Most of the above-mentioned methods for fabricating superhydrophobic-superhydrophilic patterns are complex and costly, many of which involve multiple time-consuming steps and the use of masks. The usage of masks always includes two additional steps for adding and removing the mask layer, which eventually increases the complexity and processing time. However, application of wettability patterned surfaces in real-world manufacturing requires a large area, high throughput, and low cost processing. To further advance such contrasting wettability interfaces, it is important to develop a process that can provide a sharp transition of wettability from one pattern to another as well as the capability of treating large complex surfaces at a rapid rate with the ability of automation.

In recent years, laser-based surface fabrication strategies have become very prominent processing methods for creating surface structures due to high precision, process flexibility, and ease of automation. Vorobyev and Guo employed an expensive femtosecond laser for making materials superwicking through surface nano/microstructuring.^12,29,40,41^ Their laser-based method achieved consistent superwickability for glass,^40^ silicon,^41^ noble metal,^29^ human enamel, and a dentin surface.^12^ They fabricated hierarchical surface structures with porous nanostructures on the peaks and valleys of parallel open microgrooves for gold and platinum.^29^ The nanostructures comprised nanocavities and nanoprotrusions created by laser irradiation-induced melting, splashing, and resolidification. Vertical methanol wicking was reported to be up to 24 mm along the microgrooves at a speed of 1 cm/s. However, a high capital cost associated with femtosecond lasers makes the process very expensive. Most of the recent literature on superwicking surface fabrication only investigated the surface structure generation, although the wettability at the solid–liquid interface is a function of surface chemistry and surface topography. Very little work has been devoted to the role of both surface topography and chemistry on the wicking behavior. The author’s group has recently shown feasibility of a standalone superwicking surface using a nanosecond laser.^42^

In this work, a superhydrophobicity-guided superwicking surface was fabricated on aluminum alloy 6061 (AA6061) alloy using nanosecond laser and surface chemistry modification. Surface fabrication does not require the use of masks, and it independently controls surface structures and surface chemistry of contrasting wetting regions. The superhydrophobic region had nanostructures with fluorocarbon chemistry, whereas the superwicking region possessed microgrooves covered with micro/nanostructures with polar nitrile chemistry. The vertical superwicking activities of water and methanol were captured with a camera. The transport velocity and height of methanol and water for different designs and different orientations of the processed surface were evaluated. Thus, this work is critical to understanding the wickability of laser-textured patterned wetting surfaces and providing the critical roles of surface chemistry and surface structures on wicking dynamics.

## 2 Experiment

AA6061 samples were cleaned in a sonication bath for 15 min in acetone, ethanol, and deionized (DI) water sequentially before the nanosecond laser-based high-throughput surface nanostructuring (nHSN) process. Finally, after rinsing in DI water, the samples were dried for 24 h at room temperature. The nHSN process^43−46^ was first employed to fabricate a nanostructured superhydrophobic surface on AA6061. This process included two successive steps: (i) water-confined nanosecond laser texturing (wNLT) and (ii) chemical immersion treatment (CIT). wNLT confines the laser-induced plasma during each laser pulse resulting in surface-enhancing shock peening effects. During the wNLT step, the AA6061 surface was irradiated by an infrared Nd:YAG nanosecond pulse laser (Spectra-Physics Quanta-Ray Lab-150, wavelength of 1064 nm) under water confinement in a zig-zag scanning path at a scanning speed of 3 mm/s. The intensity of the laser beam has Gaussian distribution. During the wNLT process, pulse duration was kept at 8 ns with a 10 Hz repetition rate, and pulse energy was kept at 340 mJ with a 1.5 mm beam diameter and a 75% overlap ratio. The estimated laser fluence was 19.2 J/cm^2^. A three-axis galvanometer laser scanner (SCANLAB intelliSCAN 20 and varioSCAN*_de_* 40i) with an f-theta objective lens was used for laser scanning. Due to the limitation of the experimental laser processing setup, the maximum size of the sample that can be fabricated was 100 mm × 100 mm. The laser-irradiated surface was immersion-treated for 3 h in an ethanolic solution of the 1.5% 1*H*,1*H*,2*H*,2*H*-perfluorooctyltrichlorosilane [CF_3_(CF_2_)_5_(CH_2_)_2_SiCl_3_] reagent, also known as FOTS (98%, Sigma-Aldrich). The solution was magnetically stirred for 20 min at room temperature to make sure that the solution was uniformly mixed. Subsequently, the AA6061 samples were cleaned with DI water and dried using compressed nitrogen. Later, specimens were kept in a vacuum oven at 80 °C for 1 h.

Designated superwicking regions were fabricated on the superhydrophobic AA6061 surface by nanosecond laser texturing in air (aNLT), which irradiated the surface with the same nanosecond laser but with a 120 ns pulse duration. The experiments were performed in ambient conditions at the focal plane where the beam diameter was 100 ± 12.8 μm. The estimated laser fluence was kept between 382 and 1146 J/cm^2^ by varying the laser power from 0.3 to 0.9 W. Unidirectional microgrooves were formed by scanning the laser beam from the start point to the end point with a zig-zag scanning path at a scanning speed of 0.75 mm/s. The line spacings (*l*_s_) between microgrooves were preset through the computer control. After the aNLT step, the samples were cleaned with compressed nitrogen followed by cleaning in a sonication bath using ethanol and DI water sequentially. Then, the samples were dried with compressed nitrogen. After the aNLT step, the AA6061 specimens were immersion-treated for 3 h in an ethanolic solution with 1.5% of the 3-cyanopropyltrichlorosilane reagent [CN(CH_2_)_3_SiCl_3_], also known as CPTS (97%, Sigma-Aldrich). The solution was magnetically stirred for 20 min at room temperature to make sure that the solution was uniformly mixed. Later, the specimens were cleaned using DI water and compressed nitrogen and dried in a vacuum oven at 80 °C for 1 h. The details of the process sequence are shown in Figure S1.^62^ The CPTS treatment does not significantly affect the previously treated superhydrophobic surface as will be explained in Section 3.2.

Hitachi S-4800 scanning electron microscopy (SEM) was used to analyze the surface morphology of the nHSN-treated samples. A non-contact 3D laser scanning confocal microscope (Keyence VK-X1000) was used to analyze the roughness and 3D surface profile, especially the information about the width, depth, and shape of microgrooves. Surface chemistry was analyzed using a Kratos Axis ultrahigh-performance X-ray photoelectron spectroscopy (XPS) system. Survey scans and core-level spectrum analyses were performed. More details about the settings of XPS analysis can be found in Wang et al.’s work.^45^ XPS spectra analyses were performed using the CasaXPS software. Both survey and core-level spectra were calibrated with respect to the C 1s peak at 285.0 eV.

The static contact angle (θ) was measured at ambient temperatures on the superhydrophobic and superwicking regions using a contact angle goniometer (Rame-Hart model 100). A 4 μL water droplet was micropipetted on the treated surface, and the shadowgraph was captured at an equilibrium state using a high-resolution CMOS camera (6–60× magnification, Thor Laboratories). For each specimen, an average value of six θ_w_ measurements was reported by analyzing the shadowgraph using opensource ImageJ software. For the superhydrophobic region, droplet roll-off tests were performed in an in-house designed roll-off angle (θ_Roll-off_) measurement apparatus with a resolution of 0.1°. The θ_Roll-off_ is the critical angle at which a 4 μL water droplet began to slide down on the tilted rotary stage of the apparatus. To characterize the dynamic interaction of water with the treated surface over time, a 5 μL DI water droplet was dropped on the superhydrophobic and superwicking region and water-repelling and spreading were captured using a black and white IDT X-StreamVision XS-3 CCD camera with a 50 mm lens (Nikon AF Micro-Nikkor-F/1.4) at 1000 fps (see Figure S2a). LED lights were used for back-illumination for droplet imaging. The wicking measurement was performed on an in-house designed setup, as schematically shown in Figure S2b. The specimen was clamped on a rotary stage, which can provide a specific orientation for a measurement. A continuous liquid source was provided to the bottom of the specimens, and the vertical movement was captured by a Nikon D7100 camera with a Nikkor AF-S DX 18-105MM-F/3.5 lens at 30 fps. LED lights were used for illumination of the surface for imaging of the wicking liquid front.

## 3 Results and Discussion

**Figure 1:**
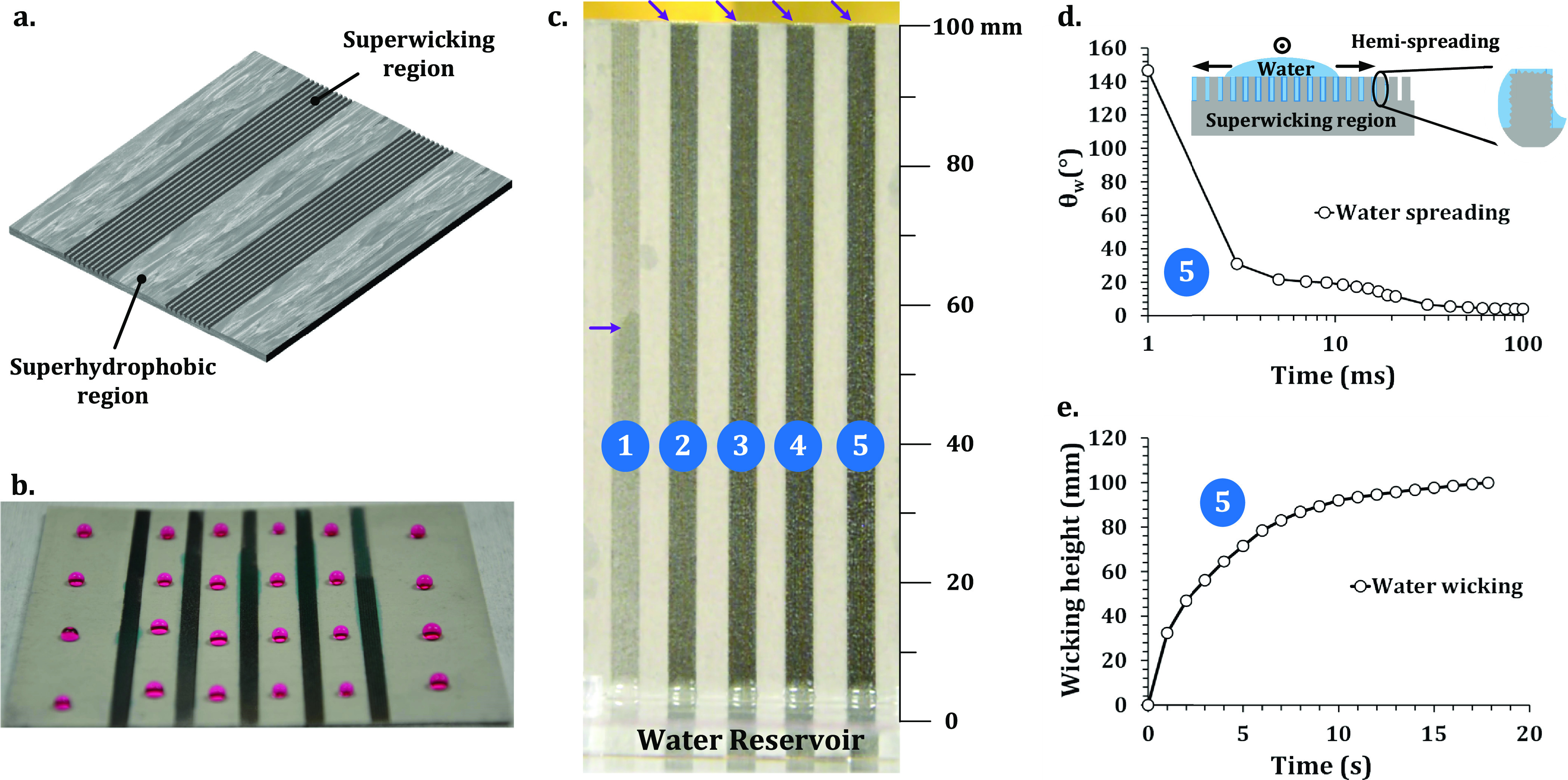
Superhydrophobicity-guided superwicking metal surface fabrication. (a) Schematic representation of the design for the superhydrophobic-superwicking patterned wetting surface. (b) Fabricated superwicking regions over a superhydrophobic background. (c) Anti-gravity superwicking effect showing that water runs vertically uphill on the designated superwicking regions for at least 100 mm. (d) Hemispherical spreading of a water droplet across and along the microgrooves. (e) 100 mm anti-gravity wicking within less than 20 s.

Using the four-step laser-chemical surface treatment, alternate superwicking and superhydrophobic patterns have been created on AA6061 alloy, as schematically shown in Figure 1a. Designated superwicking regions (4 mm × 100 mm) were fabricated on the superhydrophobic background for alternate wetting patterns. When blue liquid droplets were dropped on the superwicking patterns, they spread instantly, whereas the pink liquid droplets formed spherical droplets on the superhydrophobic regions (Figure 1b). Once the fabricated surface is tilted vertically, the spherical droplets rolled off the superhydrophobic regions, and the superwicking regions remained wet. The superhydrophobic regions have θ_w_ in the order of 158° ± 1.9° and θ_Roll-off_ in the order of 7.8° ± 0.5°. Figure 1c shows the anti-gravity superwicking effect of the laser-treated flat AA6061 plate. The arrow marks indicate the position of the waterfront on the designated superwicking regions 1–5 that correspond to laser powers of 0.3, 0.45, 0.6, 0.75, and 0.9 W, respectively. It can also be observed that the waterfront was confined in the superwicking regions by the surrounding nonwetting superhydrophobic regions. Therefore, the water transport only happened through the superwicking regions, and the remaining areas remained nonwetted. One of the objectives of the current work is to restrict the sideways flow and allow the wicking activity in the vertical direction. As the superhydrophobic surface has a very low surface energy, it stopped the sideways flow and guided the self-propelling liquid front to flow in the vertical direction. Depending on the design of surface topography and chemistry, the wickability can be controlled in the designated superwicking regions.

When a 5 μL DI water droplet was dropped on the superhydrophobic regions of the treated surface, the droplet kept bouncing on the surface, demonstrating extremely nonwetting behavior (see Movie S1). On the contrary, when a 5 μL DI water droplet was dropped on the superwicking regions of the treated surface, the droplet spread and the waterfront propagated through the superwicking region resulting in a rapid decrease in θ_w_ down to 10–5° within 12 ms of contacting the surface, as shown in Figure 1d. The droplet spontaneously spread on the surface and completely absorbed in the processed surface after 100 ms. Bico et al.^60^ demonstrated this phenomenon as hemi-spreading, as shown in Figure 1d, and it happens due to the wetting transition from the Wenzel state to the surface film state resulting from the small intrinsic θ_w_ between the micro/nanostructures and liquid interface.^61^ Water flows along the microcapillary and overflows into the neighboring microgroove through the porous micro/nanostructures of the ridges. It can also be observed that the spreading of the water droplet increases with the increase in the depth (*D*_g_) of microgrooves (see Figure S3a). Microgrooves with increasing *D*_g_ can accommodate more volume and transport more water along the grooves resulting in faster dispersion of liquids. An increasing rate of hemi-spreading of a water droplet was also observed for wicking regions with decreasing *l*_s_ between microgrooves (see Figure S3b). When the *l*_s_ was reduced, there were more microgrooves in a specific area than the larger *l*_s_. Therefore, the liquid droplet had more neighboring microgrooves for spreading. When the bottom of the treated surface was immersed in a water reservoir in vertical orientation, water runs vertically uphill through the superwicking regions (see Movie S1) for at least 100 mm within less than 20 s from the reservoir (Figure 1e).

The next three sections will study the properties of the microgrooves and their effect on the wicking behaviors. A sample with a constant *l*_s_ of 250 μm will be used in Section 3.1 to examine the morphology of the ridges and grooves, while Section 3.2 looks at the effect of surface chemistry. In Section 3.3, the effect of the microgroove geometry on the wicking behavior will be studied using a constant *l*_s_ of 250 μm as a function of laser power and then fixing the laser power at 0.6 W while varying the *l*_s_.

### 3.1 Morphology of the Patterned Wetting Surface

**Figure 2:**
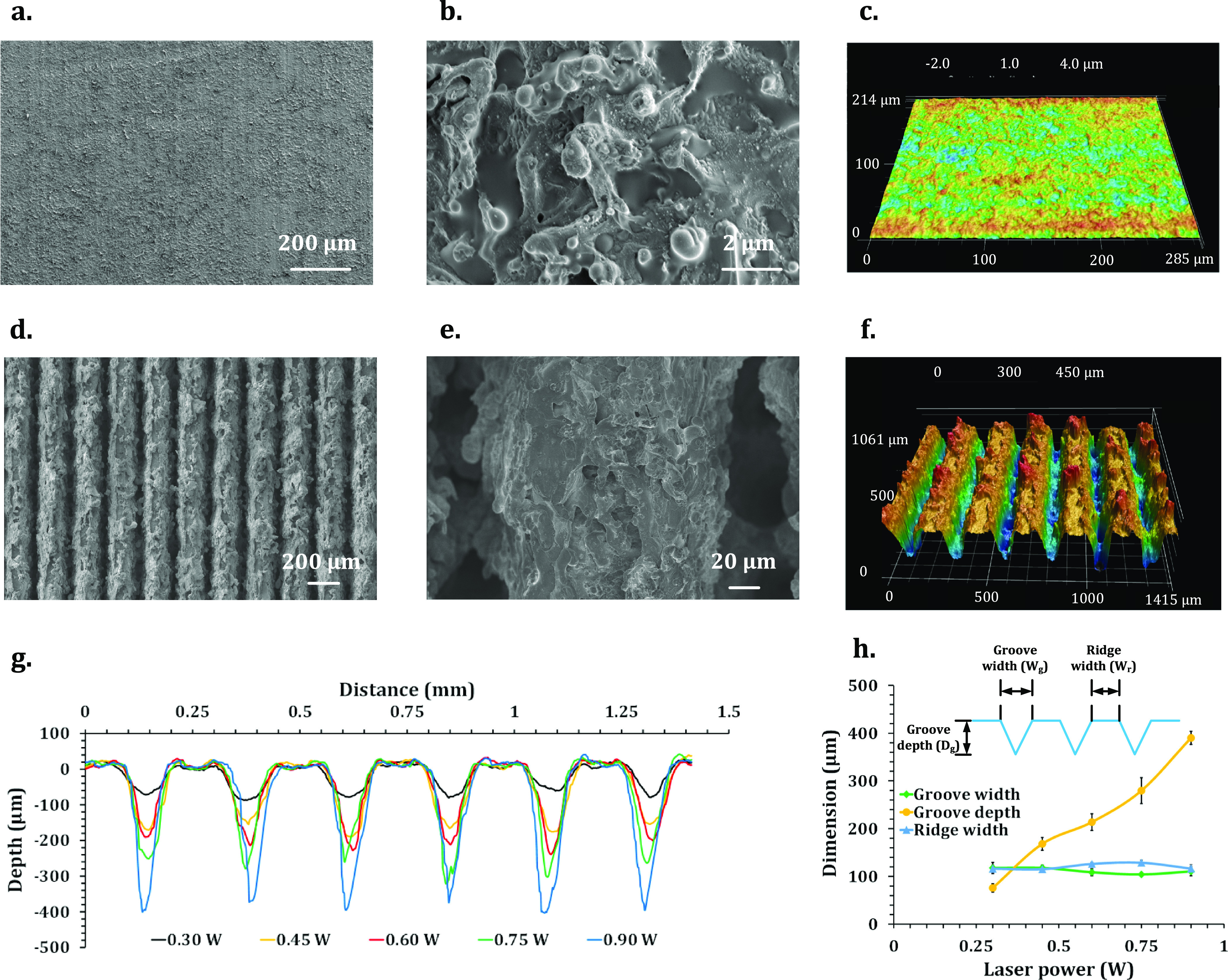
Surface topography of the patterned superwicking-superhydrophobic surface of the sample in Figure 1. (a) Isotropic, nanoscale texture in the superhydrophobic region. (b) Nanoscale surface features of ripples and pores. (c) Areal profile of the nanotextured superhydrophobic region using confocal microscopy. (d) Microgroove texture in the superwicking region. (e) Micro/nanoscale features are covering the valleys and ridges of microgrooves. (f) Areal profile of the microgroove-textured region using confocal microscopy. (g) Variation of the microgroove profile for different powers. (h) Variation of *W*_g_, *D*_g_, and *W*_r_ as a function of laser power.

The superhydrophobic regions possessed a smooth, isotropic texture, but no obvious microscale patterns can be observed (Figure 2a). At 10,000× magnification, the SEM micrograph revealed closely packed nanoscale surface features of ripple protrusions, particles, and pores, ranging in size from few tens of nanometers to several hundred nanometers (Figure 2b). As illustrated in Figure 2c, confocal microscopy further confirmed the smooth isotropic surface texture with an average roughness of 828 ± 29 nm. The superwicking region shows an array of periodically spaced microgrooves with a width of about 100 μm (Figure 2d). As illustrated in Figure 2e, randomly distributed porous micro/nanostructures enclosed the ridges and valleys of the microgrooves. Primarily, laser-induced melting, splashing, resolidification, and redeposition of molten metal caused the formation of porous micro/nanostructures on the ridges and valleys of microgrooves (see Figure S4). The presence of porous micro/nanostructures is necessary for superwicking functionalization by increasing the permeability of the surface. Capillary pressure increases with high permeability leading to a superior wicking effect.^5^ Additionally, the chemical treatment with CPTS also influenced the surface morphology change.^42^ The microgroove topography can be described by an areal profile scan using confocal microscopy, as shown in Figure 2f. A different laser power was used with the same beam diameter, scanning speed, and spacing between scanning lines. Increasing the power fabricated deeper microgrooves, as illustrated in Figure 2g. The cross section of the microgrooves is a triangular shape. With the increase in power from 0.3 to 0.9 W, the *D*_g_ of the microgrooves increased from 76 ± 9 to 390 ± 15 μm. However, the groove width (*W*_g_) at the open end and the ridge width (*W*_r_) between microgrooves remained almost constant (Figure 2h).

### 3.2 Effects of Surface Chemistry

**Figure 3:**
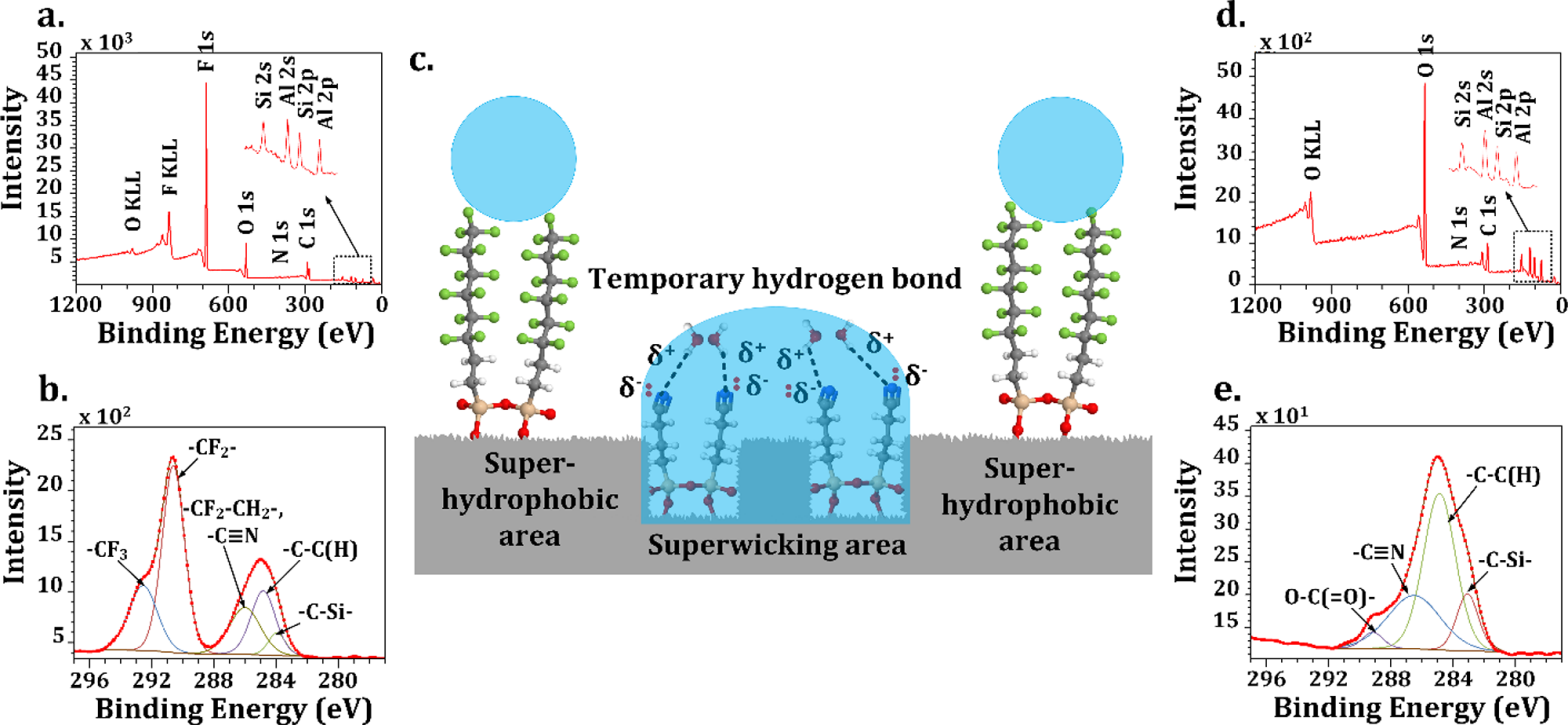
Surface chemistry analysis on the patterned superwicking-superhydrophobic surface: (a) XPS survey analysis on the superhydrophobic region. (b) Core-level XPS analysis of C 1s at the superhydrophobic region. (c) Schematic representation of water interaction in the superwicking and superhydrophobic regions. (d) XPS survey analysis on the superwicking region. (e) Core-level XPS analysis of C 1s at the superwicking region.

To analyze the surface chemistry of the superhydrophobic and superwicking regions, XPS analysis was performed on both superhydrophobic and superwicking regions. The survey spectrum shows that Al, Si, O, C, F, and N elements were present on the superhydrophobic regions (Figure 3a). AA6061 contains Al and Si as elemental composition, and O came from the surface oxidation of the specimen during laser texturing in the air. C and F signals came from −CF_3_, −CF_2_–, and −CH_2_– functional groups in the FOTS reagent [CF_3_(CF_2_)_5_(CH_2_)_2_SiCl_3_], which was used during the first chemical immersion treatment. There is also a minuscule amount of nitrogen present in the superhydrophobic region. The presence of nitrogen derives from CPTS [CN(CH_2_)_3_SiCl_3_] during the second chemical immersion treatment; however, the amount of —C≡N attached to the surface was too small in comparison with −CF_3_ and −CF_2_– functional groups to alter the wettability of the area. Core-level analysis further confirms the presence of all the above functional groups inside the C 1s peak (Figure 3b). The survey spectrum shows that Al, Si, O, C, and N elements were present on the superwicking regions (Figure 3d). However, there is no F peak present in the superwicking region. This is because the attached FOTS molecules were removed during laser-selective laser texturing in air resulting in a breakdown of silane molecules due to instantaneous extreme heat generated at the laser-material interaction zone. This incidence of the N peak was also in accordance with the molecular structure of the CPTS reagent. Core-level analysis further confirms the presence of the —C≡N group inside the C 1s peak (Figure 3e). Although both the FOTS and CPTS reagent contained three chlorine atoms in their chemical structure, there was no chlorine (Cl) signal detected in the survey spectra of superhydrophobic and superwicking regions. These chlorine atoms reacted with the aluminum substrate and dissolved in the chemical solution as aluminum chloride resulting in an etching effect during the two immersion treatments. This etching effect enhanced the porous structures in the superwicking region and nanostructures in the superhydrophobic regions.^42,45^

The nitrile group possesses very strong permanent dipole–dipole attractions and strong polarity.^47^ Conversely, water is also a polar molecule as the electronegative oxygen atom draws electrons of the covalent bond creating H^+^ and OH^–^ ions. As the surface nitrile group is also polar in nature, the delta-negative nitrogen atom in the −CN group attracts the delta-positive hydrogen atom from a water molecule. Therefore, an attraction between opposite charges creates a temporary hydrogen bond, as schematically shown in Figure 3c. The nitrile group demonstrates the capability of forming a temporary hydrogen bond, especially with water and alcohol.^48^ Furthermore, surfaces with dual-scale structures are known to enhance wicking behavior through capillary action by letting a liquid flow through microgrooves, while the small-scale surface features maintain the capillary pressure. The combination of affinitive surface chemistry and favorable surface microgrooves results in having a higher affinity toward water molecules leading to superwickability. Similarly, methanol also has hydrogen bonding capabilities and high polarity due to the existence of the −OH group in its molecular structure. Therefore, it is also expected to show capillary wicking in the fabricated surface. On the other hand, the presence of fluorocarbon groups (−CF_2_– and −CF_3_) is known in surface chemistry to reduce the dispersive component of surface energy.^49−51^ As a result, the surface behaves as a repellent to water molecules. The combination of repellent surface chemistry and surface nanostructures induces extreme repellency toward water molecules leading to superhydrophobicity. This contrasting interaction of water molecules with superhydrophobic and superwicking regions is schematically shown in Figure 3c.

**Figure 4:**
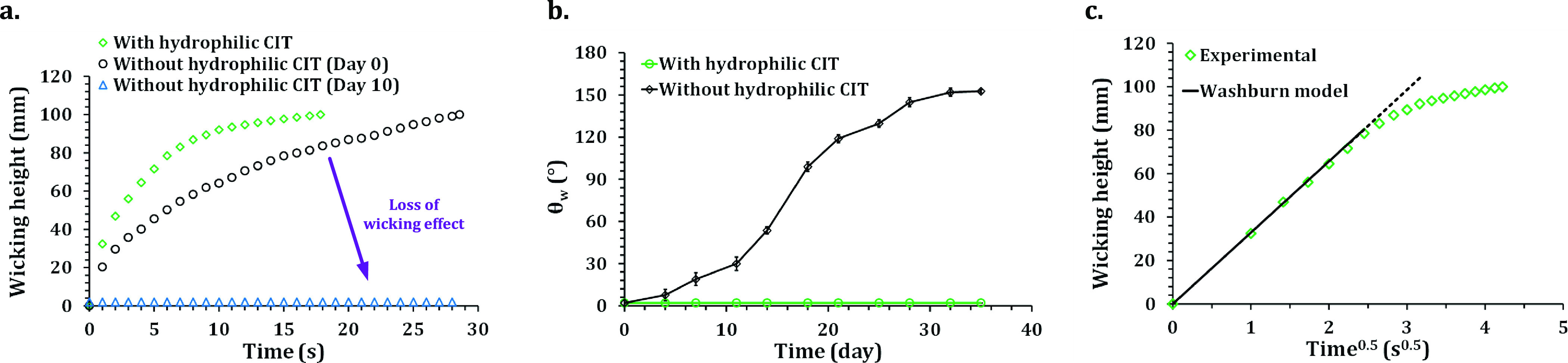
Wicking activity measurement. (a) Comparison of wicking dynamics of water between surfaces with and without hydrophilic chemical immersion treatment. (b) Stability of θ_w_ on the selectively laser-textured surface with and without hydrophilic chemical immersion treatment. (c) Plot of the vertical wicking distance marked by wetting front versus *t*^0.5^.

Since the water affinitive nitrile group was attached on top of fabricated capillary systems resulting in intrinsic superwickability, the fabricated AA6061 surface was expected to boost the capillary effect. Immediately after selective laser texturing in air, the surface was also superwicking but did not have the nitrile surface chemistry. When the wicking distance of water was compared between the fabricated wicking region with hydrophilic chemical treatment and the wicking region immediately after the laser texturing process (without hydrophilic chemical treatment), wicking enhancement can be seen, as shown in Figure 4a. Additionally, surfaces after the laser texturing process and without hydrophilic chemical treatment completely lose their wickability within 10 days of processing. The hydrophilic CIT region maintained its properties on day 10 as explained below.

As laser processing in air is a high-energy fabrication and aluminum is highly reactive to oxygen, atmospheric oxygen reacts with aluminum and produces aluminum oxide on top of textured surface structures. Due to the formation of oxides, aluminum atoms at the surface are electron-deficient and form a hydrogen bond with interfacial water molecules resulting in hydrophilicity.^52^ The presence of unsaturated oxide polar sites on the substrate induces wicking capability.^53^ However, this superwickability was not sustainable, and the wettability transited to hydrophobicity due to exposure to ambient conditions. Within 30 days, θ_w_ became 152°, as shown in Figure 4b. As the exposure to ambient conditions did not change the micro/nanostructures, this wettability transition was influenced by surface chemistry change.^54,55^ The wettability transition was caused by the adsorption of organic constituents and the absorbance of airborne hydrocarbon contamination. Adsorbed organic constituents reduced the surface energy, causing a gradual change to hydrophobicity. On the other hand, the capability of creating temporary hydrogen bonds by the −CN group was believed to have contributed toward retaining superwickability.

The capillary rise of liquid inside a closed tube is a very well-known scientific event. However, it is a less known fact that the capillary rise can also happen in a half-tube or a channel. Therefore, a strong capillary effect can be generated over a large area of a surface by carving a series of parallel microgrooves on a metal surface. It can create a superwicking surface where the capillary effect can draw the water molecule through the microgrooves. However, the self-propelling capillary force generated in a half-tube is less than a fully enclosed tube.^56^ The liquid flow dynamics inside a wicking structure is demonstrated by the Washburn equation:
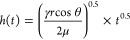
1where *h*(*t*) is the vertical distance traversed by the liquid at time *t*, γ is the liquid surface tension, μ is the liquid viscosity, *r* is the capillary radius, and θ is the contact angle*.* Wicking activity follows Washburn dynamics on an open V-shaped groove where the wicking distance is proportional to the square root of time.^57^ In this research, the microgrooves created were also open triangular-type, as illustrated in morphological analysis. Therefore, experimentally measured data were compared with the wicking rate predicted by an analytical model inspired by Washburn dynamics for perfectly ordered microgrooves to evaluate the dynamics of wicking activity on the processed surface,^57^ as seen in eq 2
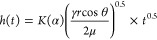
2where *K*(α) is a function influenced by the permeability of porous micro/nanostructures, groove angle, and surface chemistry.^5,57^ The capillary radius (*r*) was determined from the area and perimeter of the 2D cross-sectional profile of the microgrooves. Previously, self-propelling liquid flow following Washburn dynamics and driven by the capillary effect has been shown on surfaces with open microgrooves,^40,41^ surfaces with 2D arrays of micropillars,^58^ and tubes under microgravity conditions.^59^ As described in the Section 3.1, the dimensions of cross-sectional profiles of fabricated open microgrooves fell within the capillary size requirement for Washburn dynamics. Washburn dynamics was followed by the fabricated superwicking regions for the initial phase of wicking as the model prediction is very close to the experimentally measured values (Figure 4c). After that, the wicking effect entered the non-linear regime, and the wicking rate decreased.

### 3.3 Effect of Microgroove Geometry on Wicking Transport

**Figure 5:**
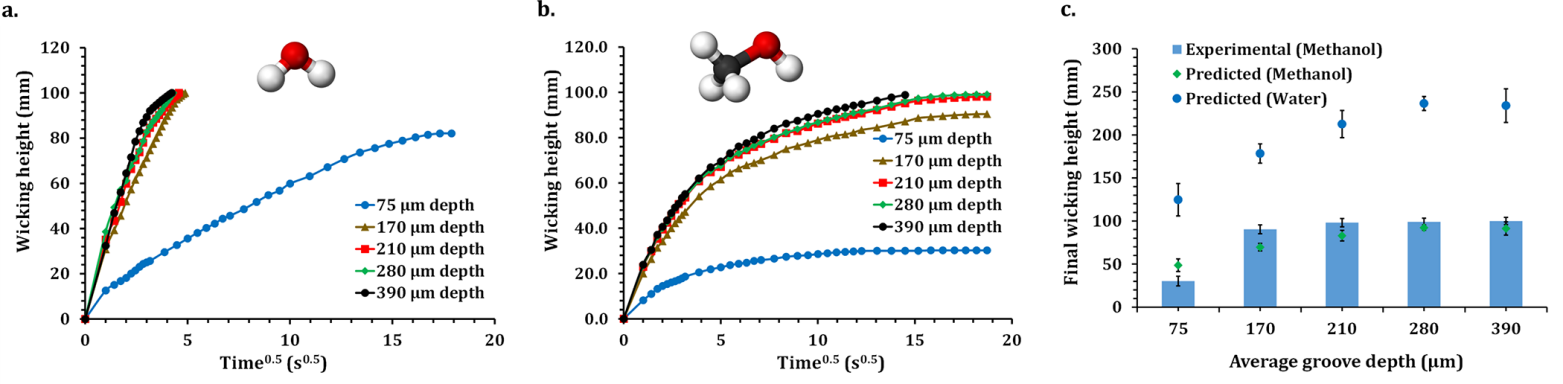
Variation of wicking transport depending on the microgroove depth. (a) Wicking rate of water to reach 100 mm height. (b) Wicking rate of methanol to reach a steady-state height. (c) Comparison of the experimentally measured and predicted final wicking height of methanol and the predicted value of water.

In this study, the *l*_s_ between microgrooves was kept constant at 250 μm while the laser power was varied from 0.3 to 0.9 W. For water wicking, the wicking region with ∼75 μm *D*_g_ had a wicking height of 82 mm after reaching steady-state conditions. All other four microgroove depths reached 100 mm wicking height of the fabricated surface (see Movie S2). The wicking rate is also observed to be dependent on the *D*_g_ of the microgroove (Figure 5a). For water wicking, the waterfront reaches 100 mm height within 18 ± 0.6 s for grooves with a *D*_g_ of ∼390 μm, whereas it takes 24 ± 0.4 s for regions with 170 μm *D*_g_. For the wicking region with 75 μm *D*_g_, it took 320 s to reach a steady-state height of 82 mm. For methanol wicking, wicking regions with increasing *D*_g_ had increasing wicking height. A similar trend was observed for the methanol wicking rate, where the wicking rate increased with increasing *D*_g_ (Figure 5b). However, the wicking rate was slow compared to water. The wicking performance difference comes from the property of the two liquids. As indicated in eq 2, the wicking rate is proportional to the square root of (γ/μ). In this case, γ and μ of methanol (γ_methanol_ = 0.0227 N/m; μ_methanol_ = 0.000594 Pa/s) are smaller than water (γ_water_ = 0.0727 N/m; μ_water_ = 0.00089 Pa/s). Water has a γ/μ ratio 2.14 times higher than methanol, resulting in an increased wicking rate.

Considering the entire cross-sectional area of the microgrooves filled with liquid, the capillary rise equation for the triangular cross section is given by^21^
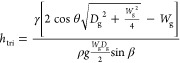
3where ρ is the density of the liquid and *g* is the gravitational constant. The microgrooves are vertically positioned (or with a slanted angle β) to facilitate the bottom end that is in constant contact with a big liquid reservoir. Heat transfer is assumed to be negligible as the capillary rise is an instantaneous rapid event.

The variation of wicking height due to the change of *D*_g_, *W*_g_, θ, β, and γ can be investigated using eq 3. In this work, we investigated the effect of *D*_g_ and γ of two different liquids on wicking height and validated against the experimentally measured wicking heights. However, due to the limitation of the laser processing setup, the maximum size of the wicking region was 100 mm. Based on the geometry measured experimentally, the theoretically predicted values of wicking height from eq 3 for methanol show a similar trend and match well with the experimentally measured values (Figure 5c). Based on the theoretical calculation, the water wicking height would have been ∼250 mm for the fabricated wicking region. However, only a 100 mm water wicking height could be measured due to the limitation of the maximum length of the fabricated wicking region. Based on the theoretical analysis, Figure S5 shows the effect of *D*_g_ at a different *W*_g_, effect of *W*_g_ at a different *D*_g_, and effect of contact angles on the wicking height of water.

**Figure 6:**
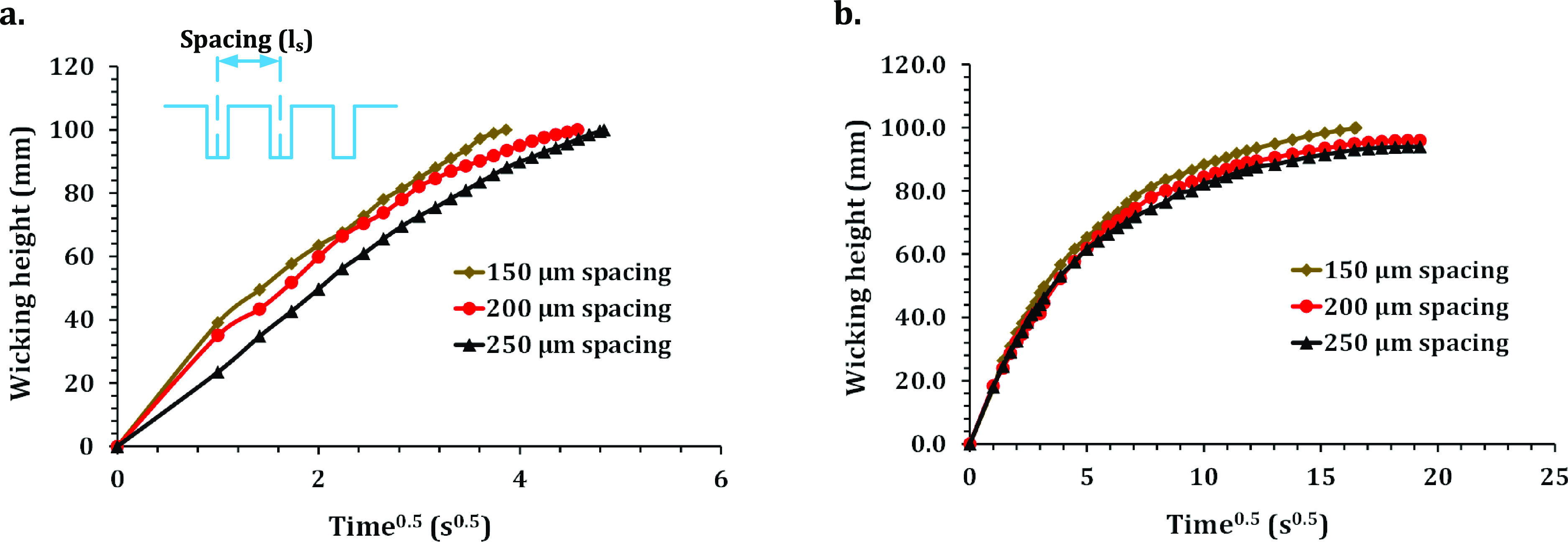
Variation of wicking transport with microgroove line spacing. (a) Experimentally measured progression of the wicking front of water. (b) Experimentally measured progression of the wicking front of methanol.

In this study, the *l*_s_ between microgrooves was varied from 250 to 150 μm while the laser power was kept constant at 0.6 W. Therefore, the average *D*_g_ of microgrooves was 213 ± 21 μm, as shown in Figure 2h. For water wicking, all specimens reached 100 mm wicking height. However, the region with a 150 μm *l*_s_ reached there faster at 14.9 s, whereas the region with a 250 μm *l*_s_ reached there slower in 23.4 s (Figure 6a). A similar trend was observed for methanol wicking (Figure 6b). For a 150 μm *l*_s_, methanol reached 100 μm wicking height in 272 s, whereas for a 250 μm *l*_s_ specimen, it only reached 94 mm wicking height at 370 s.

## 4 Conclusions

In this study, a patterned superwicking-superhydrophobic surface is fabricated to demonstrate a superhydrophobicity-guided wicking activity. Superwicking regions contain laser-irradiated microgrooves covered with micro/nanostructures with nitrile chemistry, whereas superhydrophobic regions possess nanostructures with fluorocarbon chemistry. Dual-scale microgrooves and nitrile surface chemistry with a high dipole moment enhance the wicking activity. The fabricated superwicking regions demonstrate spreading through the hemi-spreading event and superwicking dynamics by pumping water and methanol vertically from a liquid reservoir. Classical Washburn dynamics was followed during the initial phase of the wicking rise, where the wicking height is proportionate to the square root of wicking time. Later, it enters a non-linear wicking regime. Water runs vertically uphill, defying gravity on the fabricated superwicking microgrooves for at least 100 mm within less than 20 s. The wicking rate and height are dependent on the geometry of the microgrooves and agree well with the analytical prediction. Decreasing spacing between microgrooves also improves the wicking rate and height. The knowledge gained from this study will provide direction to engineer future surfaces for heat pipes, microcooling systems, thermal devices, and microfluidic channels.

## Supporting Information Available

Process sequence to fabricate the superhydrophobicity-guided superwicking surface for AA6061 alloy, schematic illustration of the setup to capture water spreading and wicking dynamics, time-dependent evolution of θ_w_ during water spreading, ridge surface showing a porous nature, and the effect of microgroove depth and width and contact angle on the theoretical wicking height of water (PDF)
Water droplet bouncing in the superhydrophobic region and vertical water wicking in the superwicking region (MP4)
Comparison of wicking rate with increasing microgroove depth (MP4)

## ABBREVIATIONS

nHSN: nanosecond laser-based high-throughput surface nanostructuring
wNLT: nanosecond laser texturing in water
aNLT: nanosecond laser texturing in air
CIT: chemical immersion treatment
θ: static contact angle
θ_Roll-off_: roll-off angle
*l*_s_: line spacing
*W*_g_: microgroove width
*W*_r_: ridge width
*D*_g_: microgroove depth
*r*: capillary radius
β: slanted angle
γ: surface tension
μ: viscosity
ρ: density
*g*: gravitational constant
*h*(*t*): wicking height
