# Supplementary material for: Enabling Superhydrophobicity-Guided Superwicking in Metal Alloys via a Nanosecond Laser-Based Surface Treatment Method
Source: ACS Appl Mater Interfaces. 2021 Aug 20;13(34):41209–19. doi: 10.1021/acsami.1c09144 (PMC8414485; doi:10.1021/acsami.1c09144)
Supplement: Supplementary file 1 — am1c09144_si_001.pdf [file am1c09144_si_001.pdf]

Enable Superhydrophobicity Guided Superwicking in Metal alloys via a Nanosecond Laser-based Surface Treatment Method

Avik Samanta[1], Wuji Huang[1], A.S.M. Sazzad Parveg[1], Parth Kotak[1], Raymond C Y Auyeung[2], Nicholas A. Charipar[2], Scott K. Shaw[3], Albert Ratner[1], Caterina Lamuta[1], and Hongtao Ding[1*]

[1]Department of Mechanical Engineering, University of Iowa, Iowa City, IA 52242, USA
[2]U.S. Naval Research Laboratory, 4555 Overlook Ave., SW, Washington, DC 20375, USA
[3]Department of Chemistry, University of Iowa, Iowa City, IA 52242, USA

[*]Corresponding author: hongtao-ding@uiowa.edu, +1 319 335 5674

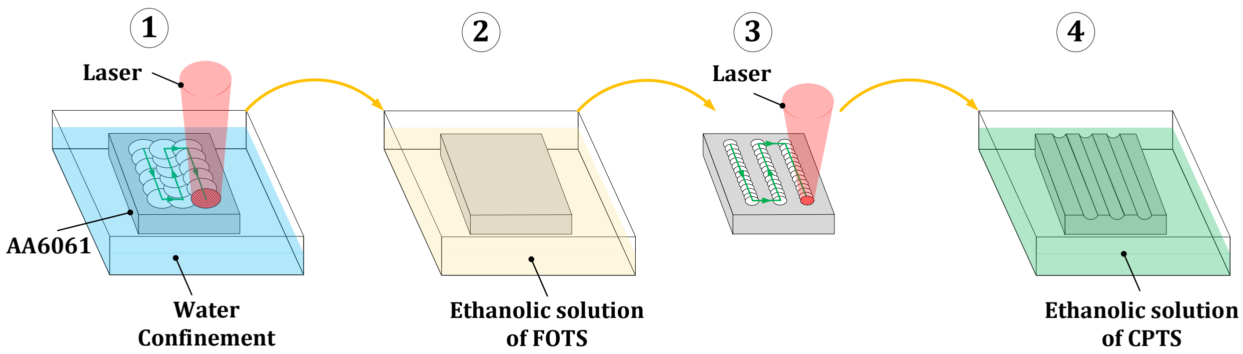

Figure S1. Process sequence to fabricate superhydrophobicity guided superwicking Surface for AA6061 alloy: (1) laser texturing under water confinement, (2) chemical immersion treatment in a ethanolic solution of FOTS, (3) selective laser texturing in air, (4) chemical immersion treatment in a ethanolic solution of CPTS.

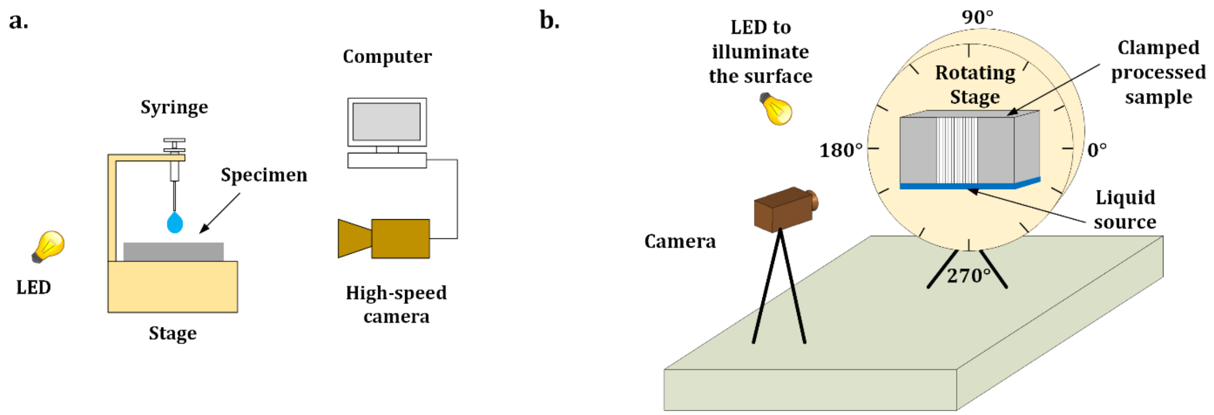

Figure S2. Schematic illustration of the setup to capture (a) water spreading and (b) wicking dynamics.

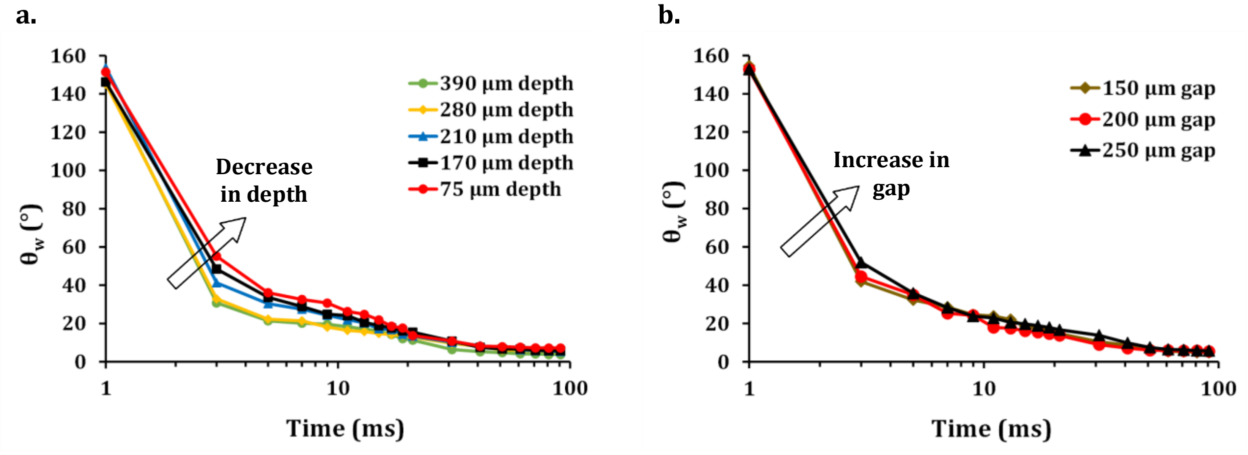

Figure S3. The time-dependent evolution of θ_w during water spreading. (a) For different microgroove depth and (b) For different microgroove spacing.

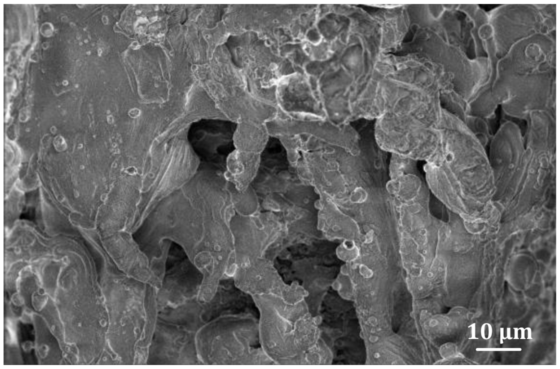

Figure S4. Ridge surface showing porous nature.

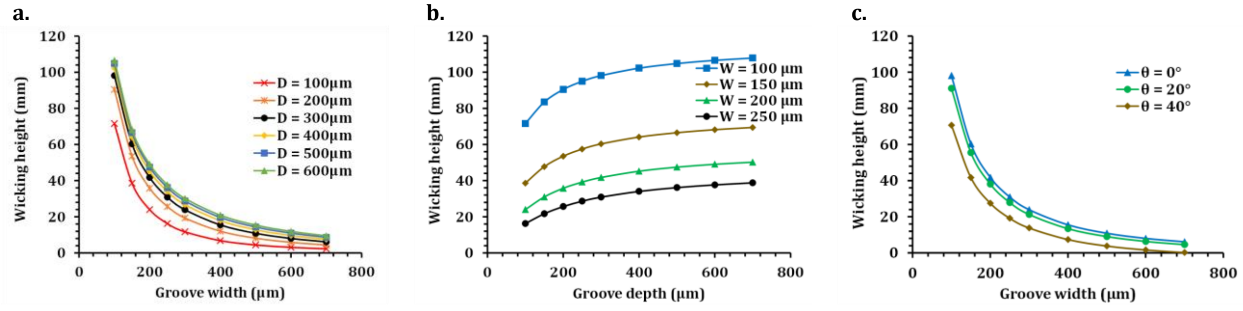

Figure S5. The effect of microgroove depth and width and contact angle on theoretical wicking height of water. (a) Effect of depth at different width. (b) Effect of width at different depth. and (c) Effect of contact angle at different width.

Movies Catalogue

Movie S1: Water droplet bouncing in superhydrophobic region and vertical water wicking in superwicking region.
Movie S2: Comparison of wicking rate with increasing microgroove depth (L-R, 75, 170, 210, 280 and 390 µm).
